# Acute heat stress amplifies exercise‐induced metabolomic perturbations and reveals variation in circulating amino acids in endurance‐trained males

**DOI:** 10.1113/EP090911

**Published:** 2023-01-24

**Authors:** Samuel Bennett, Franck Brocherie, Marie M. Phelan, Eve Tiollier, Elodie Guibert, Antonio J. Morales‐Artacho, Paul Lalire, James P. Morton, Julien B. Louis, Daniel J. Owens

**Affiliations:** ^1^ Research Institute of Sport and Exercise Science (RISES) Liverpool John Moores University Liverpool UK; ^2^ Laboratory Sport Expertise and Performance (EA 7370) French Institute of Sport Paris France; ^3^ NMR Metabolomics Shared Research Facility Technology Directorate University of Liverpool Liverpool UK; ^4^ French Triathlon Federation (FFTri) Saint Denis La Plaine France

**Keywords:** endurance exercise, heat stress, metabolism, metabolomics, triathlon performance

## Abstract

Using untargeted metabolomics, we aimed to characterise the systemic impact of environmental heat stress during exercise. Twenty‐three trained male triathletes (${\dot{V}}_{O_{2}\mathrm{peak}}$ = 64.8 ± 9.2 ml kg min^−1^) completed a 30‐min exercise test in hot (35°C) and temperate (21°C) conditions. Venous blood samples were collected immediately pre‐ and post‐exercise, and the serum fraction was assessed via untargeted ^1^H‐NMR metabolomics. Data were analysed via uni‐ and multivariate analyses to identify differences between conditions. Mean power output was higher in temperate (231 ± 36 W) versus hot (223 ± 31 W) conditions (*P* < 0.001). Mean heart rate (temperate, 162 ± 10 beats min^−1^, hot, 167 ± 9 beats min^−1^, *P* < 0.001), peak core temperature (*T*_rec_), core temperature change (Δ*T*_rec_) (*P* < 0.001) and peak rating of perceived exertion (*P* = 0.005) were higher in hot versus temperate conditions. Change in metabolite abundance following exercise revealed distinct clustering following multivariate analysis. Six metabolites increased (2‐hydroxyvaleric acid, acetate, alanine, glucarate, glucose, lactate) in hot relative to temperate (*P* < 0.05) conditions. Leucine and lysine decreased in both conditions but to a greater extent in temperate conditions (*P* < 0.05). Citrate (*P* = 0.04) was greater in temperate conditions whilst creatinine decreased in hot conditions only (*P* > 0.05). Environmental heat stress increased glycolytic metabolite abundance and led to distinct alterations in the circulating amino acid availability, including increased alanine, glutamine, leucine and isoleucine. The data highlight the need for additional exercise nutrition and metabolism research, specifically focusing on protein requirements for exercise under heat stress.

## Abstract (highlights)

- **What is the central question of this study?**
  Whole‐body substrate utilisation is altered during exercise in hot environments, characterised by increased glycolytic metabolism: does heat stress alter the serum metabolome in response to high intensity exercise?
- **What are the main finding and its importance?**
  Alongside increases in glycolytic metabolite abundance, circulating amino acid concentrations are reduced following exercise under heat stress. Prior research has overlooked the impact of heat stress on protein metabolism during exercise, raising important practical implications for protein intake recommendations in the heat.

## 1 INTRODUCTION

Ambient temperatures exceeding 30°C significantly impair endurance exercise capacity (Peiffer & Abbiss, 2011; Periard & Racinais, 2016). The physiological impact is multifactorial (Sawka et al., 2011) and encompasses cardiovascular, neurological, psychological and metabolic alterations, the magnitude of change ranging from 5% to 20% dependent on environmental temperature, humidity, exercise intensity and the subject's hydration status (Gonzalez‐Alonso et al., 1997; Sawka et al., 2011).

Environmental heat stress significantly alters substrate metabolism during exercise (Febbraio, 2001), with numerous studies reporting increased carbohydrate utilisation with a concomitant decrease in lipid oxidation during exercise (Febbraio et al., 1994a; Hargreaves et al., 1996). These metabolic permutations are characterized by an elevated respiratory exchange ratio (Febbraio et al., 1994; Hargreaves et al., 1996), hyperglycaemia (Febbraio et al., 1994, 1994; Hargreaves et al., 1996), increased blood and muscle lactate (Febbraio et al., 1994a; Parkin et al., 1999) and elevated muscle glycogenolytic rate (Febbraio et al., 1996; Fernandez‐Elias et al., 2015; Parkin et al., 1999). Based on this metabolic understanding, traditional exercise nutrition guidelines have advocated high carbohydrate intake pre‐, during and post‐exercise in hot ambient conditions to support the increased rate of carbohydrate utilisation and facilitate muscle glycogen resynthesis between exercise bouts (Burke, 2001).

The impact of heat stress on exercise metabolism has traditionally been investigated via targeted metabolite analysis, including glucose, lactate and other small metabolically generated molecules (ATP and phosphocreatine, along with increases in ADP, AMP, ammonia, inosine 5′‐monophosphate) (Febbraio, 2001). Recent advancements in analytical chemical techniques, including the application of mass spectrometry and nuclear magnetic resonance (NMR) spectroscopy in the field of exercise metabolism (Sakaguchi et al., 2019) have facilitated the global characterisation of metabolite concentrations within biofluids in response to exercise. Principally, metabolomics permits an exploratory insight into underlying metabolic processes during exercise spanning multiple functional groups including carbohydrate metabolites and amino acids. Metabolomics techniques have been implemented in the characterisation of altitude/hypoxia on the exercise metabolome (Lawler et al., 2019; Messier et al., 2017), highlighting increases in glycolysis, amino acid and purine metabolism compared to normoxic/sea level exercise. Importantly, circulating amino acids are crucial precursors for protein synthesis (Gwin et al., 2020), and decreased availability following exercise may lead to sub‐optimal recovery from exercise (Millard‐Stafford et al., 2008; Tipton & Wolfe, 2001). The impact of environmental conditions on amino acid availability warrants further investigation to ensure appropriate recovery strategies are advised. Lacking evidence of altered amino acid metabolism during exercise under heat stress has led to comprehensive carbohydrate‐centric nutritional recommendations for exercise in the heat (Burke, 2001). Whether protein intake requirements should be adjusted following exercise in hot conditions remains unresolved.

The present study aimed to characterise the serum metabolome during exercise under environmental heat stress to gain greater insight into the metabolic impact of environmental heat stress on exercise metabolism and highlight potential metabolites of interest specific to exercise in hot conditions.

## 2 METHODS

### 2.1 Ethical approval

All participants provided written informed consent, and all procedures conformed to the standards of the latest revision of the *Declaration of Helsinki*, except for registration in a database. Ethical approval was granted by the Comité de Protection des Personnes Ouest IV–Nantes (CPP) (No. 2018‐A02544‐51).

### 2.2 Study design

Implementing a within‐subjects repeated measures design, participants completed two exercise capacity tests in hot (35°C, ∼50% relative humidity (RH)) and temperate (21°C, ∼50% RH) conditions in a randomised order separated by 2 days. In the week preceding experimental trials, subjects completed a thorough familiarisation of all procedures and testing protocols. All subjects replicated their dietary intake for the 24 h before any laboratory visit and were instructed to refrain from alcohol or caffeine consumption and strenuous physical activity in the 48 h before each battery of tests. Approximately 2 h before arriving at the laboratory, subjects consumed a standardised pre‐exercise meal (CHO: 2.0 g kg body mass (BM)^−1^; PRO: 0.3 g kg BM^−1^; fat: 0.3 g kg BM^−1^) that was provided by the research team alongside comprehensive written instructions and guidelines.

### 2.3 Participants

Twenty‐four trained male triathletes (29 ± 7 years, 72.9 ± 6 kg, 181 ± 6 cm) with a mean peak oxygen consumption (${\dot{V}}_{O_{2}\mathrm{peak}}$) and maximal aerobic power (MAP) of 62.3 ± 6.6 ml kg min^−1^ and 330 ± 40 W, respectively, were recruited for this study. Participants were not acclimated to heat stress, and to account for seasonal variation in environmental temperatures, testing procedures were halted during the summer months (June–September).

Participants underwent comprehensive medical screening and examination by an onsite physician and were free of musculoskeletal or neurological disease and not under any pharmacological treatment during the study. One person withdrew from the study, citing personal reasons.

### 2.4 Preliminary testing: assessment of maximal oxygen uptake

In the week preceding the study (∼7 days), ${\dot{V}}_{O_{2}\max}$ and MAP were assessed via an incremental cycle test performed on an electronically braked cycle ergometer (Excalibur Sport, Lode, Groningen, Netherlands) as previously described (Impey et al., 2015). Briefly, following a 10‐min warm‐up at 100 W, workload was increased in 30 W increments ever 2 min until volitional failure. Breath‐by‐breath oxygen uptake (${\dot{V}}_{O_{2}}$) and carbon dioxide uptake (${\dot{V}}_{CO_{2}}$) were obtained throughout the exercise using an online gas analysis system (Quark, Cosmed, Rome, Italy). End‐point criteria for ${\dot{V}}_{O_{2}\max}$ being achieved were: (1) heart rate within 10 beats min^−1^ of age‐predicted maximum, (2) respiratory exchange ratio >1.1, and (3) plateau of oxygen consumption despite increased workload. MAP was calculated as per Hawley & Noakes (1992).

### 2.5 Thirty‐minute capacity test (hot and temperate conditions)

Participants completed a 30‐min cycling capacity test in hot (35°C, 50% RH) and temperate (20°C, 50% RH) conditions in a randomised order (Kim & Shin, 2014) matched for time of day, and separated by at least 2 days. A fixed‐duration exercise test with intermittent sprints was implemented to reflect the demands of Olympic distance triathlon cycling (Etxebarria, D'Auria et al., 2014). The tests were conducted on participants’ bicycles mounted on a previously validated stationary cycle home trainer (Hammer H2, CycleOps, Fort Lauderdale, FL, USA) (Lillo‐Bevia & Pallares, 2018).

#### 2.5.1 Exercise protocol

**FIGURE 1:**
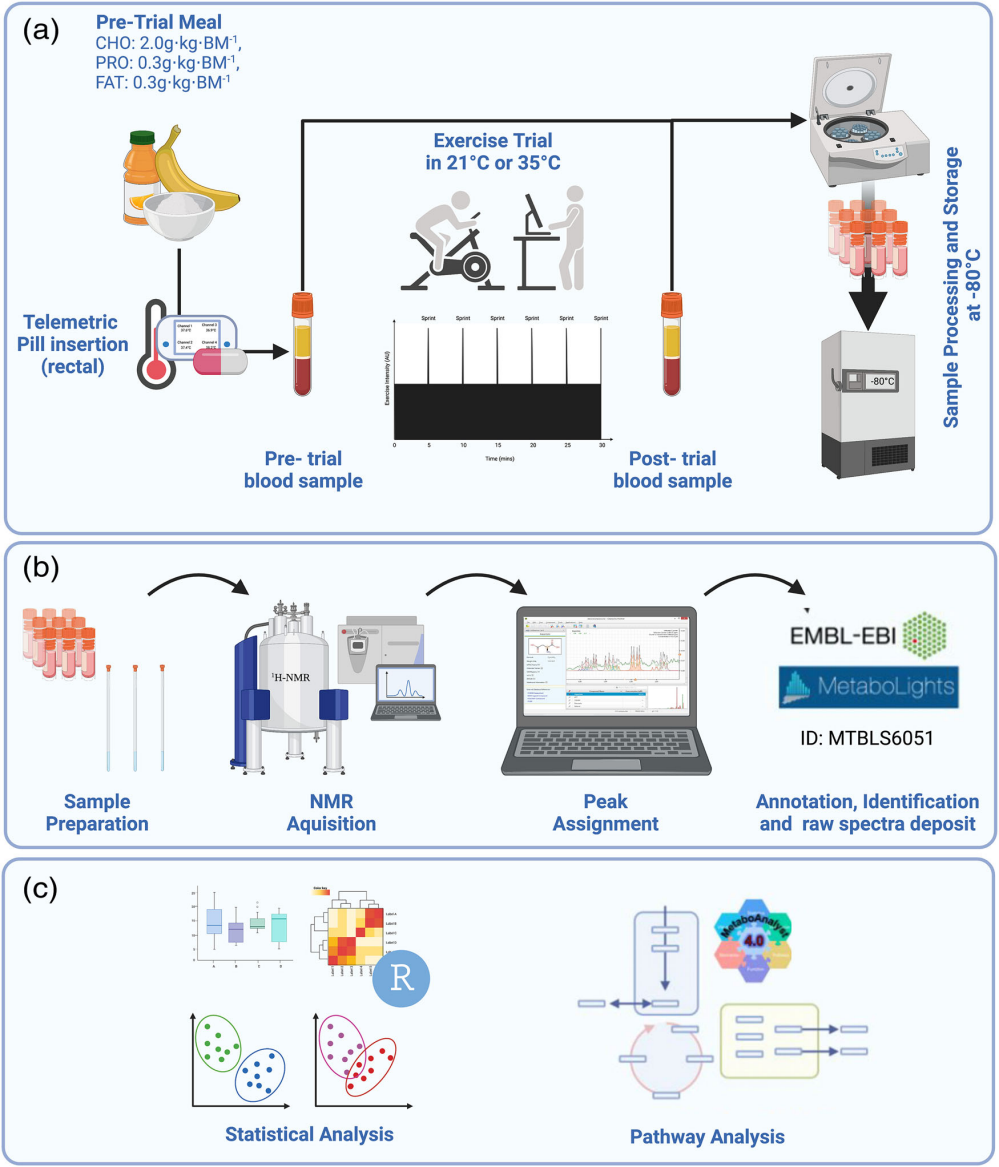
Schematic overview of the study. (a) Test day protocol commenced with a pre‐exercise breakfast ∼2 h prior. One hour before exercise, subjects inserted a telemetric pill rectally before a pre‐exercise blood sample was collected. Subjects completed a 15‐min warm‐up before the commencement of the exercise test. Upon completion of the test, a post‐exercise sample was collected, and all samples were processed and stored immediately. (b) Sample preparation and ^1^H‐NMR analysis with peaks assigned using Chenomx; the entire parameter set raw and processed spectra are deposited in the EMBL‐EBI repository MetaboLights. (c) Univariate and multivariate analyses of metabolomics data were performed in R, and pathway analysis was completed using MetaboAnalyst. Created with BioRender.com.

Following a 15‐min self‐paced warm‐up (replicated between trials), subjects completed the test consisting of six phases of 4 min 50 s interspersed with a 10‐s sprint resulting in 30 min in total duration (Figure 1a). Participants were instructed that the aim of the test was to obtain the highest mean power (including sprints) possible during the 30 min. Live data were available to the participants, including power output, heart rate (HR), cadence, and duration remaining of the test and the current phase (Mookycentre, Paris, France).

#### 2.5.2 Core temperature

Core temperature (*T*_rec_) was measured via a telemetric pill (E‐Celcius, BodyCap, Hérouville Saint‐Clair, France). To standardise location and remove potential contamination from fluid intake during exercise, a validated suppository approach was selected and the pill was inserted beyond the anal sphincter approximately 1 h before exercise (Gosselin et al., 2019). Data were recorded at 1‐min intervals via a data logger (e‐Viewer, BodyCap, Hérouville Saint‐Clair, France).

#### 2.5.3 Perceptual measures

Fifty seconds before each sprint (approximately every 5 min), participants were provided subjective rating of perceived exertion (RPE) (6–20 Borg scale), and thermal sensation and comfort using a modified visual scale previously described (Zhang et al., 2004).

#### 2.5.4 Blood collection

Blood samples were collected from an in‐dwelling cannula in the superficial anti‐cubital fossa. Samples were collected in serum separator tubes (BD Biosciences, Plymouth, UK), stored at room temperature for 30 min, before centrifugation at 1500 *g* for 15 min at 4°C as per the manufacturer's instructions. Serum was aliquoted into cryovials and stored at −80°C until analysis (Figure 1a).

### 2.6 ^1^H‐NMR metabolomics

#### 2.6.1 Sample preparation

Previously aliquoted serum was thawed and diluted at 50% (v/v) with 10% NMR buffer (^2^H_2_O with 100 mM sodium phosphate buffer pH 7.4 and 0.1% azide) before centrifugation for 5 min at 21,500 *g* and 4°C. Six hundred microlitres of the centrifuged sample was pipetted into 5 mm (outer diameter) glass SampleJet NMR tubes (Bruker, Coventry, UK).

#### 2.6.2 ^1^H‐NMR set‐up and acquisition

All spectral acquisition used a Bruker 700 MHz Avance III HD spectrometer equipped with a TCI cryoprobe and chilled autosampler (Bruker, Ettlingen, Germany). Standard vendor pulse sequences were applied to collect 1D ^1^H‐NMR spectra (cpmg1dpr). A Carr–Purcell–Meiboom–Gill (CPMG) edited pulse sequence was employed to attenuate signals from macromolecules present (proteins, etc.). Serum spectra were collected at 37°C with 32 transients for optimal sensitivity, with all other parameters constant. Parameter sets along with raw and processed spectra were deposited in the EMBL European Bioinformatics Institute (EBI) repository MetaboLights (MTBLS6051).

#### 2.6.3 Spectral processing and quality control

All spectra were pre‐processed at the spectrometer by Fourier transformation, phase correction and baseline correction using standard vendor routines (apk0.noe) and referenced against anomeric glucose signal (serum). Spectra were subject to quality control criteria as recommended by the Metabolomics Standards Initiative (MSI) (Salek et al., 2013; Sumner et al., 2007), comprising an appraisal of baseline, line‐width, residual water signal width, phase and signal‐to‐noise. Spectra were bucketed according to peak boundaries defined with each bucket, the sum of the integral for that region divided by the region width.

#### 2.6.4 Metabolite annotation and identification

Metabolites were annotated via metabolite recognition software Chenomx (Chenomx v 8.2, Chenomx Ltd, Edmonton, Alberta, Canada), and the respective buckets were annotated before statistical analysis. Metabolite identities were confirmed (where possible) by comparison with an in‐house metabolite library. Whilst the entirety of the serum metabolome is measured by ^1^H‐NMR spectroscopy, adherence to strict quality control, identification and reporting guidelines significantly reduced the number of metabolites presented compared to unvalidated mass spectrometry approaches.

### 2.7 Statistical analysis

Performance and physiological variables, including mean power output, mean HR, Δ*T*_rec_ and peak RPE were analysed with a paired Student's *t*‐test whilst core temperature, RPE, thermal comfort, and thermal sensation were analysed by two‐way ANOVA, using GraphPad Prism (Version 9.3.1, GraphPad Software, San Diego, CA, USA). Cohen's *d* coefficient for effect size was calculated and referenced against benchmarks suggested by Cohen (1988), where the thresholds for *d* to be considered small, medium or large are <0.2, 0.21–0.79 and >0.8, respectively.

Metabolomics analyses were performed using the statistical software R (version 4.1.1) and RStudio (Version 1.4.1) (Le Cao et al., 2017) with scripts provided by the Computational Biology Facility (CBF) at the University of Liverpool (UK). All spectra were normalised and scaled by probabilistic quotient normalisation (PQN), and Pareto (with mean scaling) approaches, respectively, due to the robustness of PQN in the analysis of complex biofluids (Kohl, Klein et al., 2012) and the optimal ability to identify small biologically significant variations in metabolites (Smolinska et al., 2012).

Metabolomics analysis was divided into two distinct approaches. Firstly, exploratory global cross‐sectional analysis was conducted whereby all available samples were included in the analysis to identify the distinct metabolic signatures from pre‐ to post‐exercise in hot and temperate conditions. Samples from pre‐ and post‐exercise and environmental conditions were analysed via two‐way ANOVA. *P*‐values were adjusted for false discovery rate using the Benjamini–Hochberg method, with adjusted *P*‐values of >0.05 considered significant. Tukey's *post hoc* analysis provided pairwise comparisons between conditions and time points. Differences between environmental conditions were appraised using multivariate analysis, including principal component analysis (PCA) and partial least squares‐discriminant analysis (PLS‐DA). The number of components required for each model generated via PLS‐DA was calculated via 50 times five‐fold cross‐validation using 70% of the data (training data). The remaining 30% of the data were used to test the model's accuracy (test data).

To gain further insight into metabolite level differences following exercise, the same metabolomic workflow was implemented, albeit only matched samples with pre‐ and post‐exercise samples were included. The inclusion of matched within‐subjects samples allowed for the calculation of relative metabolite abundance difference (ΔAbundance) following exercise. The requirement for paired samples ultimately reduced the sample size to *n* = 13 in hot and *n* = 11 in temperate conditions with 10 subjects represented in both environmental conditions.

## 3 RESULTS

### 3.1 Performance and physiological data

**FIGURE 2:**
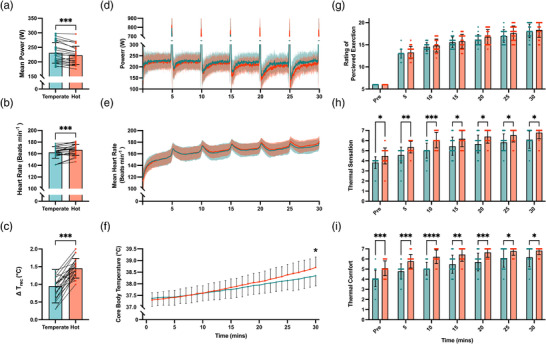
Performance and physiological responses during the 30‐min capacity test in hot (35°C, 50% RH) and temperate (21°C, 50% RH) conditions. (a–f) Mean power output (W) (a), mean heart rate (beats min^−1^) (b), core temperature change from pre to post exercise (Δ*T*_rec_, °C) (c), second‐by‐second mean power output (d), heart rate (e) and minute‐by‐minute mean core temperature (°C) (f) during 30‐min capacity test. (g–i) Perceptual responses to heat stress during exercise recorded every 5 min including rating of perceived exertion (RPE) (g), thermal sensation (h), and thermal comfort (i). A significant difference is denoted by asterisks: *P < 0.05, **P < 0.01, ***P < 0.001, ****P < 0.0001.

Mean power output was significantly lower in hot (223 ± 31 W) than temperate (231 ± 36 W) conditions (*P* < 0.001, *d* = 0.24) (Figure 2a). Mean HR was significantly higher during exercise in hot (167 ± 9 beats min^−1^) compared to temperate (162 ± 10 beats min^−1^) conditions (*P* < 0.001, *d* = 0.53) (Figure 2b). The RPE at the end of exercise was significantly higher in hot (19 ± 2) than in temperate (18 ± 2) conditions (*P* < 0.01, *d =* 0.5). Mean *T*_rec_ was not significantly different between conditions (*P* = 0.07, *d* = 0.28); however, peak *T*_rec_ was greater in hot (38.8 ± 0.4°C) compared to temperate (38.3 ± 0.4°C) conditions (*P* < 0.0001, *d* = 1.25) reflected by increased Δ*T*_rec_ in hot (1.45 ± 0.28°C) compared to temperate (0.95 ± 0.47°C) conditions (*P* < 0.001, *d* = 1.29) (Figure 2c). ANOVA revealed increased thermal sensation (*P* < 0.0001) (Figure 2h) and comfort (*P* < 0.0001) (Figure 2i) throughout the capacity test in hot conditions, with both significantly increased throughout each exercise test (*P* < 0.0001).

Second‐by‐second mean power output (Figure 2d) and heart rate (Figure 2e) alongside 5‐min‐by‐5‐min mean core body temperature (Figure 2f) in hot and temperate conditions are reported throughout the exercise test. Due to the high sampling frequency, comparisons of power output and heart rate were limited to whole trial mean data previously analysed by *t*‐test and reported above.

### 3.2 Human serum metabolomics

**FIGURE 3:**
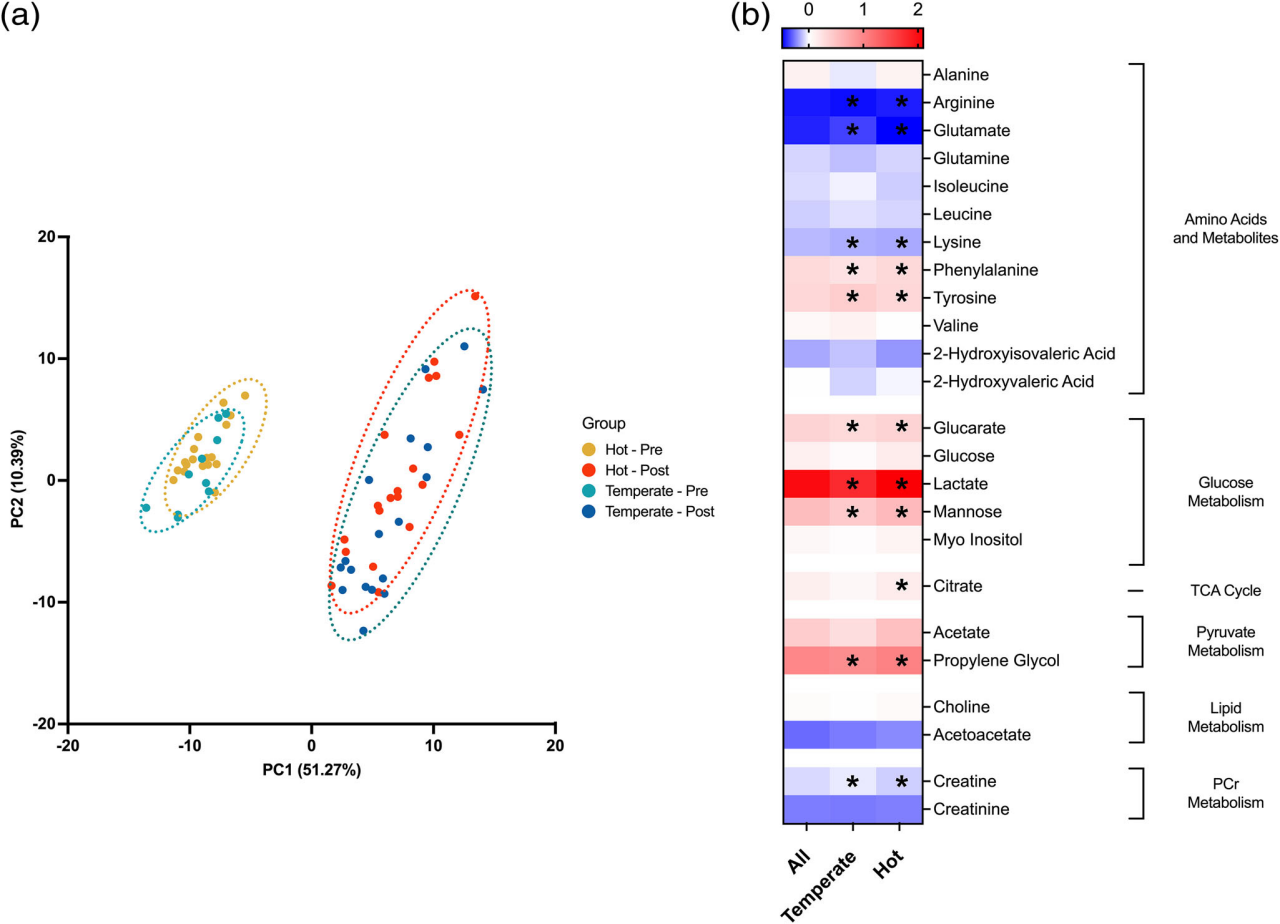
(a) Multivariate principal component analysis (PCA) of human serum pre‐ (0 min) and post‐ (30 min) exercise in hot (35°C) and temperate (21°C) conditions. Brackets represent the variance explained by PC, with 9 PCs required to explain 95% of the variance. For clarity, only PC1 and PC2 are shown on each axis. Ellipses represent a 95% confidence region. (b) Heatmap of all identified metabolites reporting the fold change from pre‐ to post‐exercise as the natural logarithm (log2) to indicate whether each metabolite level had increased (greater than 0, red) or decreased (less than 0, blue) following exercise. ‘All’ refers to the fold change of metabolites in the dataset without stratification for environmental conditions. *Significant difference in metabolite abundance from pre‐ to post‐exercise.

A total of 35 unique metabolites were identified from 172 spectral bins across the ^1^H‐NMR spectra of human serum. Where multiple bins represented one metabolite, a single representative bin for each metabolite was identified via in‐house criteria determined by correlation reliability score (Grosman, 2020). Unsupervised multivariate analysis of serum metabolomes between conditions was conducted to identify whether any underlying structures in the data were present (Figure 3a). Pre‐ to post‐exercise metabolomes were distinctly dissimilar; however, there was no apparent difference between hot and temperate conditions. Analysis by PLS‐DA revealed several metabolites critically important in the differentiation between time point and group. As with PCA, distinct clustering was observed between pre‐ and post‐exercise but no difference between hot and temperate conditions. Univariate analysis revealed no significant metabolite level differences in serum metabolome pre‐exercise, with 11 significantly different following exercise in temperate conditions, with three additional metabolites (14 in total) significantly different following exercise in hot conditions (Figure 2b and Table 1). When considering between‐groups comparisons post‐exercise, there were no significantly different metabolites between hot and temperate conditions, with only 2‐hydroxyisovalerate showing a trend toward significance in hot conditions (*P* = 0.055).

**TABLE 1:** Metabolites identified as significantly different during univariate ANOVA analysis (adjusted *P*‐value) from human serum collected immediately pre‐ and post‐exercise in hot and temperate conditions and PLS‐DA generated variance in importance of projection (VIP) scores for each metabolite.

	ANOVA			Pairwise comparisons				PLS‐DA VIP scores	
Metabolite	*F*	*P*	Adjusted *P*	Pre‐ temperate versus hot	Post‐hot versus pre‐hot	Post‐temperate versus pre‐temperate	Post‐temperate versus hot	Comp 1	Comp 2
Propylene glycol	191.06	5.81 × 10^−31^	1.51 × 10^−29^	0.3381	2.03 × 10^−11^	2.03 × 10^−11^	0.6506	1.76	1.65
Isopropanol	103.26	7.18 × 10^−24^	9.34 × 10^−23^	0.9430	2.03 × 10^−11^	2.03 × 10^−11^	0.8147	1.67	1.55
Arginine	88.23	3.70 × 10^−22^	3.21 × 10^−21^	0.9583	2.03 × 10^−11^	2.04 × 10^−11^	0.9902	1.65	1.54
Lactate	59.01	5.06 × 10^−18^	3.29 × 10^−17^	0.9754	2.04 × 10^−11^	1.80 × 10^−10^	0.9994	1.53	1.42
Mannose	47.14	6.86 × 10^−16^	3.57 × 10^−15^	0.0498	2.46 × 10^−11^	2.09 × 10^−7^	0.3386	1.57	1.51
Phenylalanine	39.95	2.08 × 10^−14^	9.01 × 10^−14^	0.9511	2.24 × 10^−10^	1.49 × 10^−8^	0.7814	1.54	1.46
Creatine	27.06	2.98 × 10^−11^	1.11 × 10^−10^	1.0000	5.24 × 10^−8^	1.06 × 10^−6^	0.9798	1.40	1.29
Tyrosine	13.15	1.00 × 10^−6^	3.25 × 10^−6^	0.9725	0.0029	0.0001	0.3983	1.15	1.13
Lysine	12.08	2.63 × 10^−6^	7.59 × 10^−6^	0.9955	0.0001	0.0028	0.9154	1.08	1.03
Glutamate	11.33	5.29 × 10^−6^	1.37 × 10^−5^	0.1782	0.0011	0.0025	0.2084	0.93	1.00
Glucarate	9.34	3.60 × 10^−5^	0.0001	0.9693	0.0003	0.0228	0.9943	0.85	0.78
Citrate	8.61	0.0001	0.0002	0.2010	0.0004	0.2794	0.9903	1.00	1.00
2‐Hydroxyisovalerate	6.90	0.0004	0.0009	0.1013	0.0725	0.1244	0.0553	0.58	1.00
Glucose	4.30	0.0081	0.0150	1.0000	0.0124	0.3798	0.6038	0.66	0.76
Acetate	3.54	0.0197	0.0341	0.9988	0.0322	0.3645	0.9078	0.61	0.60
Acetoacetate	3.43	0.0224	0.0364	0.6442	0.1404	0.2497	0.5402	0.49	0.64
Alanine	3.13	0.0320	0.0489	0.2598	0.1187	0.9357	0.9107	0.45	0.58

### 3.3 Change in serum metabolome

**FIGURE 4:**
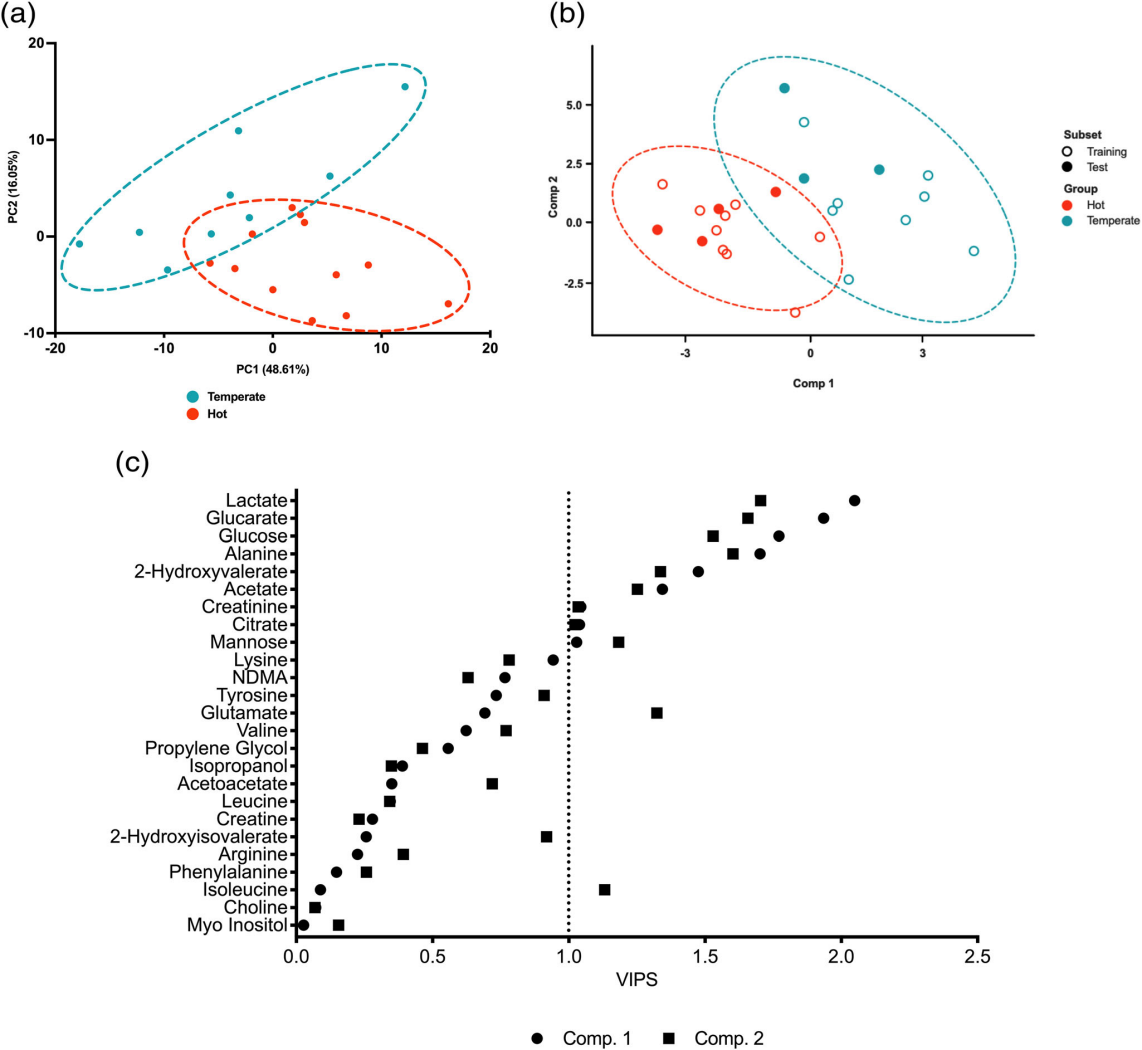
Multivariate analysis of change in human serum metabolite concentration from pre‐ to post‐exercise in hot (35°C, 50% RH) and temperate (21°C, 50% RH) conditions. (a) Principal component analysis (PCA) between environmental conditions. Brackets represent the variance explained by PC, with eight PCs required to explain 95% of the variance. For clarity, only PC1 and PC2 are shown in each panel. Ellipses represent a 95% confidence region. (b) Partial least squares discriminant analysis (PLS‐DA) scores plots representing components 1 and 2 for change in human serum metabolite concentration discriminating between hot (35°C) and temperate (21°C) conditions. Circular data points represent samples on which the model was built (training), and triangular data points represent samples used to validate the model (test). Ellipses show the 95% confidence region. (c) VIP scores of PLS‐DA models built upon intervention‐dependent differences in human serum metabolome for components 1 and 2. Circular points represent VIP scores for metabolites in component 1, and square points represent VIP scores for component 2. The vertical dashed line represents the VIP > 1 threshold.

**FIGURE 5:**
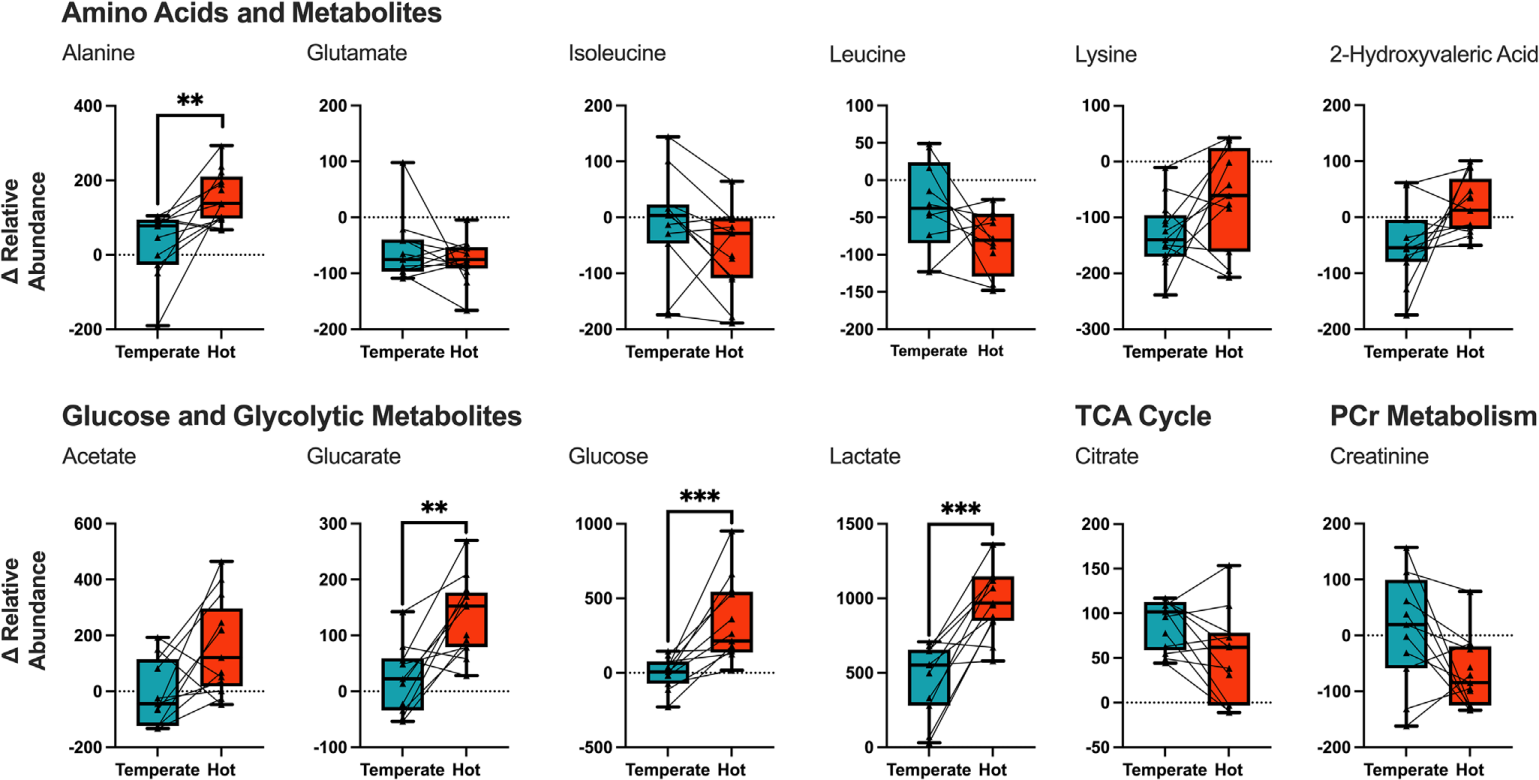
Box‐plots representing median Δ abundance from pre to post of VIP filtered serum metabolites following exercise in hot (35°C, 50% RH) and temperate (21°C, 50% RH) conditions. A significant difference between hot and temperate conditions is denoted by asterisks: **P < 0.01, ***P < 0.001.

To highlight the effect of acute heat stress on serum exercise metabolome and provide greater insight into metabolite level differences, change (Δ) in the relative abundance of each metabolite was calculated for each subject with pre‐ and post‐exercise samples in hot and temperate conditions. Multivariate PCA analysis revealed distinct underlying clustering between environmental conditions following exercise in hot and temperate conditions (Figure 4a). A two‐component cross‐validated PLS‐DA model similarly revealed distinct clustering per condition and identified 10 metabolites essential in the differentiation between groups (Figure 4b). VIP filtered metabolites (Figure 4c) from each PLS‐DA were compared via a BH adjusted *t*‐test to gain metabolite level information on condition‐specific differences; four metabolites were significantly different, including lactate, glucarate, alanine and glucose. As previously, 2‐hydroxyvaleric acid and acetate exhibited a trend toward significance but did not reach the *P* < 0.05 threshold (Figure 5).

## 4 DISCUSSION

Although the metabolic impact of acute heat stress during exercise has been relatively well defined (Febbraio, 2001), to our knowledge, the present study is the first to characterise its impact on the exercise metabolome. The application of metabolomics in exercise physiology, specifically in trained populations, is in its relative infancy, with all of the current literature being conducted in temperate conditions (Schranner et al., 2020). Here, we provide novel exercise metabolomics data related to environmental heat stress, highlighting that high‐intensity endurance exercise elicits comparable disturbances to the serum metabolome in hot and temperate conditions. Despite this, several metabolites were altered in response to exercise and heat stress when considered at the metabolite level, highlighting potential novel metabolic implications for exercise in hot conditions.

Principle component analysis revealed differences in the serum metabolome from pre‐ to post‐exercise with little difference between hot and temperate conditions (Figure 2a). Confirmed by univariate analysis, 11 and 14 metabolites were significantly different following exercise in hot and temperate conditions, respectively (Figure 2b). When considering within‐subject metabolite changes from pre‐ to post‐exercise, building a PLS‐DA model identified 10 metabolites critically important for differentiation between conditions (Figure 4a), with four significantly different between conditions following univariate analysis (Figure 5). Collectively, the present analysis highlights the relatively small contribution of heat stress to alterations in the serum metabolome during exercise in hot conditions. Nevertheless, the data reveal increased glycolytic metabolite abundances following exercise in the heat, supporting previously reported increases in glycolysis during heat stress and exercise. Additionally, the data presented here provide novel insight into the impact of heat stress during exercise on circulating amino acid concentration, highlighting potential practical implications and future research considerations.

Increased relative hyperglycaemia is consistently observed during exercise under heat stress (Febbraio et al., 1994, 1996, 1994b; Fernandez‐Elias et al., 2015). Whilst the exact source of circulating glucose is experimentally challenging to identify, increased blood glucose during exercise in hot conditions is potentially due to increased liver glucose output with minimal alterations in whole‐body glucose metabolism (Hargreaves et al., 1996). Mechanistically, altered glucose kinetics during exercise in the heat is partly due to elevated circulating plasma adrenaline, which is increased during exercise in hot conditions, inducing hepatic glucose production and increased rates of muscle glycogenolysis (Febbraio et al., 1998). Although we did not quantify circulating adrenaline here nor characterise muscle glycogen utilisation, the rationale that increased circulating adrenaline alters glucose metabolism during exercise and is amplified with heat stress is well established (Chesley et al., 1995; Febbraio et al., 1998; Howlett et al., 1999). Furthermore, during exercise in hot conditions, increases in blood glucose are accompanied by increases in circulating lactate, suggesting greater flux via anaerobic glycolysis (Febbraio et al., 1994, 1994). The metabolomic dataset provided here highlights several glycolytic metabolites, including acetate, glucarate, glucose and lactate, are increased in hot relative to temperate conditions, suggesting increased glycolytic flux in response to environmental heat stress. The aetiology of altered substrate metabolism during heat stress is multifactorial and encompasses multiple organ systems. Mechanisms include reduced oxygen provision to skeletal muscle as competition for blood flow occurs following cutaneous vasodilatation for thermoregulation (Rowell et al., 1968), greater recruitment and utilisation of fast‐twitch muscle fibres (Young et al., 1985), the direct effect of muscle temperature increasing the rate of enzyme‐mediated reactions (*Q*_10_ effect) (Young et al., 1985) and increased circulating adrenaline concentration (King et al., 1985; Yaspelkis et al., 1993). The implementation of targeted‐omics such as ^13^C‐fluxomics to determine metabolite concentrations and absolute flux through large networks would permit metabolic phenotyping of cells or tissues allowing greater elucidation of specific metabolic pathway contributions to energy provision during exercise and the origin and fate of specific metabolites of interest.

The view on lactate, historically considered a metabolic by‐product and terminal metabolite during exercise, and which contributed to fatigue and limiting exercise capacity (Brooks et al., 2022), has undergone a significant paradigm shift. It is increasingly considered a crucial metabolic intermediate functioning as an energy source, major gluconeogenic precursor and signalling molecule (Brooks, 2020). Whilst lactate in skeletal muscle is oxidised by the mitochondria (Hashimoto et al., 2006) or converted into glycogen (Hargreaves & Spriet, 2020), the metabolic fate of the lactate that is released into circulation includes utilisation as an energy substrate in the heart (Gertz et al., 1988) and brain (Glenn et al., 2015), whilst the remainder of circulating lactate will be used as gluconeogenic substrate in the liver (Bergman et al., 2000). Crucially, circulating lactate is a critical metabolic precursor for liver gluconeogenesis, and increased production during exercise in hot condition may directly or indirectly increase circulating glucose concentration in the blood; the exact mechanisms by which this occurs remain to be resolved.

Alongside increases in glycolytic metabolite abundance, alanine similarly increased during exercise and was further augmented in response to heat stress. Alanine concentrations are elevated following a bout of high‐intensity interval training (Peake et al., 2014) and exhaustive sub‐maximal exercise (Zafeiridis et al., 2016), consistent with the nature of our exercise protocol (30‐min maximal capacity test with six high‐intensity sprint intervals). Given the glucogenic role of alanine, we hypothesise that increases in circulating alanine during exercise in hot conditions are representative of the multi‐tissue alanine cycle whereby alanine is liberated at the peripheral tissue level (skeletal muscle) and transported via the bloodstream to the liver for gluconeogenesis (Felig, 1973; Felig & Wahren, 1971; Williams et al., 1998). It has also been proposed that an increase in circulating alanine with a concomitant decrease in glutamate, as observed here, is indicative of a rightward shift in the alanine aminotransferase‐catalysed reaction, a predominant anaplerotic mechanism during exercise (Gibala et al., 1997; Sahlin et al., 1990). Reductions in circulating glutamate during exercise are expected (Ahlborg et al., 1974; Felig & Wahren, 1971), with established consensus that a significant source of carbon for expansion of TCA cycle intermediate production is glutamate‐derived; as such, intramuscular glutamate concentration decreases sharply at the onset of exercise (Katz et al., 1986), leading to greater intramuscular glutamate uptake. Additionally, decreases in serum glutamate concentrations may result from increased liver uptake due to the critical role of glutamate in the alanine cycle as it is converted to alanine by alanine aminotransferase, serendipitously corroborating our reported increase in circulating alanine (Wagenmakers, 1998).

Alterations to circulating amino acid concentrations, specifically the branched‐chain amino acids (BCAA) isoleucine and leucine, have been previously reported during exercise (Ahlborg et al., 1974; Felig & Wahren, 1971). Leucine and isoleucine, represent ketogenic amino acids and are oxidised by skeletal muscle during exercise (McKenzie et al., 2000). Initially, BCAA catabolism requires the TCA cycle intermediate 2‐oxoglutarate; as such, TCA cycle intermediates are reduced during exercise when BCAA metabolism is required (Gibala et al., 2002). It is well established that protein intake following exercise increases skeletal muscle protein synthetic rates (Churchward‐Venne et al., 2020) with leucine increasingly considered a vital amino acid for the stimulation of protein synthesis following endurance exercise. Increased amino acid turnover during exercise and heat stress may have a negative impact on post‐exercise recovery and adaptation, and as such, future research should aim to elucidate the impact of heat stress on the circulating amino acids and the potential impact on recovery. As with carbohydrate recommendations for exercise and heat stress, protein requirements may also require revision.

A relatively unknown metabolite in the context of exercise is 2‐hydroxyvaleric acid; nevertheless, the increases in this metabolite's abundance may be of interest to exercise physiologists. Elevations in urinary 2‐hydroxyvaleric acid have been associated with lactate acidaemia induced by succinate acidaemia (Asano et al., 1988) and propionyl‐CoA carboxylase deficiency (Bergstrom et al., 1981). Most interestingly, increases in lactic acid, 2‐hydroxyvaleric acid and succinic acid were associated with impaired protein activities involved in succinate metabolism (succinate dehydrogenase) and mitochondrial respiratory complexes I and III. Increased 2‐hydroxyvalerate may be a consequence of increased metabolism of isoleucine, which is converted to propionyl‐CoA, a metabolic precursor of succinyl‐CoA, a TCA cycle intermediate. Due to increased glycolytic rate and a concomitant decrease in citrate abundance in the heat, excess acetyl‐CoA and propionyl‐CoA may lead to increased 2‐hydroxyvalerate and attenuation of isoleucine catabolism (Goodman et al., 1978; Sweetman et al., 1978; Stokke et al., 1973). Whilst requiring further investigation in the context of exercise in hot conditions, 2‐hydroxyvalerate may represent an important biomarker for branch chain amino acid metabolism and impaired mitochondrial oxidation during exercise.

Practically, endurance exercise increases skeletal muscle protein synthesis rates (Harber et al., 2010; Mascher et al., 2011), utilising amino acids as ‘building blocks’ for *de novo* protein synthesis. As such, to allow optimal recovery and protein synthesis rates, amino acids must be available for this process to occur. The reduction in circulating amino acids reported in the present study following exercise may lead to sub‐optimal recovery as there are insufficient amino acids available to support post‐exercise skeletal muscle protein synthetic rates. Protein intake immediately following endurance exercise has been shown to increase circulating amino acid concentrations and increase muscle protein synthesis in a dose‐dependent manner (Churchward‐Venne et al., 2020).

Notably, the samples collected pre‐exercise were not from the fasted state; instead they were from approximately 2–3 h post‐standardised meal, which coincides with peak post‐prandial gluconeogenic amino acid appearance (Bos et al., 2003), meaning circulating alanine abundance may be influenced by prior dietary intake and not solely exercise. Nevertheless, the standardisation of pre‐exercise food intake means that there is likely an equal impact of diet on each environmental condition. Therefore, the attestation that alanine is increased following exercise in hot conditions remains true. However, identifying the precise origin of circulating alanine remains challenging due to the inherent difficulty in quantifying tissue‐specific protein breakdown.

It is widely accepted that a period of heat acclimation reduces the physiological impact of heat stress and thus reduces metabolic demands of exercise (Febbraio et al., 1994). Despite this, the time course of metabolic adaptation in response to heat stress is not characterised. The potential for readily accessible capillary blood collection for metabolomics analysis (Catala et al., 2018) in conjunction with indirect measures of whole body metabolism represents a promising strategy for the elucidation of heat‐induced changes in metabolism during exercise and impact of heat acclimation on the exercise metabolome. The use of capillary blood sampling would also increase the accessibility of ‘real‐world’ data collected within the field and increase the availability of biological samples. The use of venous blood collection within this study, and in the context of exercise, led to multiple samples being omitted due to logistical constraints. The lack of a complete sample set, and high inter‐individual variability in response to exercise and heat stress impaired within‐subjects metabolomics analysis. For instance, evidence presented here potentially supports the hypothesis that a minimum temperature threshold exists, and must be achieved before alterations in metabolism are observed (Febbraio, 2001). For example, physiological data reveals blunted temperature increases (Δ*T*_rec_) in a subset of participants (Figure 2c), which may account for the irregular metabolite specific changes in some participants (Figure 5). Unfortunately, the reduced number of samples included during the within‐subjects analysis precludes further analysis to definitively understand whether this critical temperature threshold exists during exercise and heat stress.

Although a consistent and robust analytical approach (Periard & Racinais, 2016; Psychogios et al., 2011), NMR metabolomics constitutes an untargeted technique that only reports primary metabolites (Psychogios et al., 2011). Being limited to primary metabolites poses specific limitations for biological interpretation. Much of the discussion within this paper has focused on carbohydrate and amino acid metabolism with no discussion of the impact of exercise and heat stress on lipid metabolism. Few studies have investigated the impact of heat stress on lipid metabolism, with plasma free fatty acid (FFA) concentrations consistently reported unchanged during exercise in hot conditions (Fink et al., 1975; Nielsen et al., 1990; Yaspelkis et al., 1993). Critically, plasma FFA concentrations merely reflect the net difference between whole‐body lipolysis and tissue uptake during exercise. Likely due to the high levels of variability in analytical techniques, the sole literature reporting intramuscular lipid metabolism by Fink et al. (1975) reported intramuscular triglyceride utilisation was reduced in hot conditions. The application of NMR spectroscopy for metabolomics utilises a CPMG pulse train, which attenuates signals from large molecules such as proteins. Given the mechanism of FFA transportation in the blood via albumin or within chylomicrons, much of the serum lipidome data is suppressed and thus preventing investigations of the systemic lipid response to exercise, neglecting a key metabolic substrate within the exercise metabolome.

A further limitation of this study is the exclusively male population, and as such, the generalisability of the results is limited to endurance‐trained males and should not be extrapolated to females. Generally, the impact of heat stress on metabolism is widely under‐investigated in females, with no literature investigating the impact of heat stress during exercise on female metabolism. Whilst it is beyond the scope of this research, the application of serum metabolomics in the investigation of female exercise metabolism in response to heat stress could provide further insight into the endocrinological impact of menstrual phase on metabolic regulation during exercise and thermal stress.

### 4.1 Conclusion

We have provided a novel NMR metabolomics dataset in endurance‐trained males responding to exercise in hot conditions. Our data demonstrated comparable perturbations to the exercise metabolome between hot and temperate conditions. However, metabolite‐level differences were present. This dataset supports previously reported alterations to substrate metabolism, with increases in glycolytic metabolites, raises several important questions concerning the origin of glucose responsible for hyperglycaemia, and proposes novel metabolic read‐outs requiring further investigation in the context of exercise and heat stress.

## AUTHOR CONTRIBUTIONS

Samuel Bennett, Franck Brocherie, Paul Lalire, Eve Tiollier, Julien B. Louis, and Daniel J. Owens conceived and designed the study. Samuel Bennett, Franck Brocherie, Eve Tiollier, Julien B. Louis, Daniel J. Owens, E Guibert, A. Morales‐Artacho and Marie Phelan performed data collection. Samuel Bennett, Marie Phelan and Daniel J. Owens analysed and interpreted the data. Samuel Bennett and Daniel J. Owens drafted the manuscript. All authors have approved the final version of the manuscript and agree to be accountable for all aspects of the work in ensuring that questions related to the accuracy or integrity of any part of the work are appropriately investigated and resolved. All persons designated as authors qualify for authorship, and all those who qualify for authorship are listed.

## CONFLICT OF INTEREST

None.

## Supporting information

Statistical Summary Document

## Data Availability

Parameter sets along with raw and processed spectra were deposited in the EMBL European Bioinformatics Institute (EBI) public repository MetaboLights (MTBLS6051).
